# Sex-related difference in the retinal structure of young adults: a machine learning approach

**DOI:** 10.3389/fmed.2023.1275308

**Published:** 2023-12-14

**Authors:** Flávia Monteiro Farias, Railson Cruz Salomão, Enzo Gabriel Rocha Santos, Andrew Sousa Caires, Gabriela Santos Alvarez Sampaio, Alexandre Antônio Marques Rosa, Marcelo Fernandes Costa, Givago Silva Souza

**Affiliations:** ^1^Instituto de Ciências Biológicas, Universidade Federal do Pará, Belém, Brazil; ^2^Núcleo de Medicina Tropical, Universidade Federal do Pará, Belém, Brazil; ^3^Instituto de Ciências Exatas e Naturais, Universidade Federal do Pará, Belém, Brazil; ^4^Instituto de Ciências da Saúde, Universidade Federal do Pará, Belém, Brazil; ^5^Departamento de Psicologia, Instituto de Psicologia, Universidade de São Paulo, São Paulo, Brazil

**Keywords:** retina, retinal thickness, macula, machine learning, sex-related differences

## Abstract

**Purpose:**

To compare the accuracy of machine learning (ML) algorithms to classify the sex of the participant from retinal thickness datasets in different retinal layers.

**Methods:**

This cross-sectional study involved 26 male and 38 female subjects. Data were acquired using HRA + OCT Spectralis, and the thickness and volume of 10 retinal layers were quantified. A total of 10 features were extracted from each retinal layer. The accuracy of various algorithms, including k-nearest-neighbor, support vector classifier, logistic regression, linear discriminant analysis, random forest, decision tree, and Gaussian Naïve Bayes, was quantified. A two-way ANOVA was conducted to assess the ML accuracy, considering both the classifier type and the retinal layer as factors.

**Results:**

A comparison of the accuracies achieved by various algorithms in classifying participant sex revealed superior results in datasets related to total retinal thickness and the retinal nerve fiber layer. In these instances, no significant differences in algorithm performance were observed (*p* > 0.05). Conversely, in other layers, a decrease in classification accuracy was noted as the layer moved outward in the retina. Here, the random forest (RF) algorithm demonstrated superior performance compared to the others (*p* < 0.05).

**Conclusion:**

The current research highlights the distinctive potential of various retinal layers in sex classification. Different layers and ML algorithms yield distinct accuracies. The RF algorithm’s consistent superiority suggests its effectiveness in identifying sex-related features from a range of retinal layers.

## Introduction

Over the past 30 years, optical coherence tomography (OCT) has been used as a non-invasive method for image acquisition to evaluate the anterior and posterior segments of the eye in both diseased and healthy conditions of the human retinal structure (1–3). The development of eye diseases can occur throughout life due to the natural aging process, exposure to unhealthy lifestyle habits, systemic disorders, or genetic inheritance. In addition to these factors, sex-related factors, such as the concentrations of sex hormones that vary throughout an individual’s life, can also influence the development of eye diseases (4–6).

The existence of sexual dimorphism of the retina in humans has been investigated using OCT. The first findings of retinal sexual dimorphism pointed to a larger total retinal thickness in male subjects than in female subjects (7–12). However, the debate regarding retinal layers remains open, as some studies have observed that some retinal layers are thicker in male subjects than in female subjects, while other investigations have found no or few sex-related differences (13–19).

Overall, investigating sex-related features in the human retina is an important area of research that could lead to new insights into the causes of retinal diseases, the development of sex-specific treatments, and the design of more effective medical devices for the eye and the possible impact of postmenopausal hormone replacement anti-estrogenic therapy therapy (20).

Due to the large amount of data extracted from the retina during an OCT scan, the use of machine learning methods could be an alternative candidate for analyzing OCT data. Machine learning methods have been used due to their ability to capture complex relationships, work with high-dimensional data, generalize to new data, be flexible and adaptable, and perform automated learning of relevant features, reducing the need for human intervention (21–23). Compared to norms based on populational averages, which may not account for the significant individual variability that exists within each sex group, machine learning models can capture and leverage this variability, allowing for more precise and individualized assessments of retinal thickness. This individualized precision can be particularly valuable in clinical decision-making as it takes into account the uniqueness of each patient’s condition. Additionally, retinal thickness datasets can exhibit complex patterns and subtle variations that may not be fully captured by simple norm-based criteria.

In the present study, we aimed to evaluate the performance of several machine learning algorithms to predict the sex of the participants based on information from retinal structure features. Our primary goal was to identify which retinal layers are best to correctly classify the sex of the participant and which machine learning algorithms are better for predicting the participant’s sex in the different retinal layers.

## Materials and methods

### Ethical considerations

The present study was approved by the Ethical Committee for Research in Humans of the Universidade Federal do Pará (report number 3.285.557). All participants were informed about the experimental procedures and gave written consent to participate in the study.

### Participants

The sample consisted of 26 male participants (mean age ± standard deviation: 26.19 ± 4.96 years) and 38 participants (mean age ± standard deviation: 26.05 ± 4.68 years). All participants had normal visual acuity or were corrected to 20/20 visual acuity using a refractive lens. Only two participants (one male and one female) used optical corrections of −0.5 and −0.7 diopters and we considered that any imprecision of their OCT measurements had little or no influence on the results. Participants with neurological, systemic, eye, or retinal diseases that affected the structure or function of the visual system were excluded.

### OCT imaging

Retinal OCT imaging was performed using the Spectralis HRA + OCT system (Heidelberg Engineering GmbH, Heidelberg, Germany). Each session consisted of a 25-line horizontal raster scan in a 20°×20° area centered on the fovea, followed by 24 automated real-time repetitions. The Heidelberg Eye Explorer software (Heidelberg Engineering GmbH, Heidelberg, Germany) was used to segment retinal layers [total retina (TR), retinal nerve fiber layer (RNFL), ganglion cell layer (GCL), inner plexiform layer (IPL), inner nuclear layer (INL), outer plexiform layer (OPL), outer nuclear layer (ONL), and retinal pigmented epithelium (RPE)] and three combinations of retinal layers [overall retinal, outer retinal layers (ORL), which range from the external limiting membrane to Bruch’s membrane, and inner retinal layers (IRL), which range from the inner limiting membrane to the external limiting membrane]. The thickness and volume of each layer were quantified. Visual inspection of the segmentation was performed to avoid possible errors. The outcome of the image segmentation of retinal layers was the mean thickness of nine macular subfields (central, nasal inner, temporal inner, superior inner, inferior inner, nasal outer, temporal outer, superior outer, and inferior outer), following the Early Treatment Diabetic Retinopathy Study (ETDRS) grid. The volume of each layer was also extracted.

For each participant, the examination was performed by the same operator following the manufacturer’s guidelines. Two images were obtained in sequence for each eye on the same day. The first image was used as a reference to scan the same parts of the retina during the second image (device’s follow-up mode). The thickness of both images was averaged for subsequent analysis. Data were acquired from 128 eyes with the Spectralis HRA + OCT system, and 64 eyes were randomly selected for analysis.

### Machine learning algorithms

Prior to the application of ML algorithms, a bootstrap resampling method was employed, utilizing 200 replications for each feature derived from OCT readings. A total of 10 features were used for each retinal layer, comprising nine subfield thicknesses and the volume of the retinal layer. Python scripts were utilized for data analysis and normalization, feature selection, and the execution of ML algorithms through the training and testing phases. The performance of the ML was subsequently evaluated.

We utilized the *StandardScaler* function from the sklearn. Preprocessing package to standardize the features into standard deviation units, as shown in Equation 1.


$$\begin{array}{l} \mathrm{Standardized\_feature}\\ \;=\mathrm{(feature–mean)/standard\_deviation}\;\mathrm{(Equation 1)} \end{array}$$

The standardized features were used to train and test seven supervised ML algorithms:

The *sklearn.neighbors.KNeighborsClassifier* function was employed to implement the k-nearest neighbors (kNN) algorithm, utilizing the Minkowski distance and a k-value within the range of 5–10. The optimal k-value, which yielded the highest accuracy, was determined using the *GridSearchCV* function.

The support vector classifier (SVC) utilizes *sklearn.svm.SVC* function with the radial basis function kernel. The gamma and C parameters are set to 1 and 10, respectively.

The *sklearn.linear_model.LogisticRegression* function is utilized for logistic regression (LR), with the parameters “*penalty*” and “*solver*” set to “*l1*” and “*liblinear*,” respectively.

The *sklearn.discriminant_analysis.LinearDiscriminantAnalysis* function is utilized for linear discriminant analysis. The parameters “*solver*” and *“store_covariance*” are set to “*svd*” and “*true*,” respectively.

The *sklearn.ensemble.RandomForestClassifier* function is utilized in the application of random forest (RF), with the parameters set as follows: “*criterion*” is set to “*gini impurity*,” “*n_estimators*” is set to 50, and “*max_depth*” is set to 6.

The decision tree (DT) employs the *sklearn.tree. DecisionTreeClassifier* function, maintaining identical parameter values for “*criterion*,” “*n_estimators*,” and “*max_depth*,” as utilized in the RF algorithm.

Gaussian Naïve Bayes (GNB) using the *sklearn.naive_ bayes.GaussianNB* function.

The accuracy of ML algorithms in correctly classifying the data was evaluated (Equation 2).


$$\begin{array}{l} \mathrm{Accuracy}\\ \;=\mathrm{(true positives + true negatives)/total}\;\mathrm{(Equation 2)} \end{array}$$

True positives represent the data points correctly classified as male, while true negatives denote those accurately identified as female. The total refers to the overall number of data points.

The *ShuffleSplit* function from the *Scikit-learn* library (version 0.21.3) was utilized to divide the data, allocating 70% for model training and 30% for model testing.

### Statistics

We used a *t*-test to compare the thickness of the different datasets obtained from both eyes of male and female subjects and to later carry out an intergroup comparison of retinal layer thickness. We conducted a one-way ANOVA to evaluate the influence of macular field in the retinal thickness as well as two-way ANOVA to evaluate the influence of the classifier type and retinal dataset factors on the accuracies (model training and model testing) of the classifier. For multiple comparisons, we employed the Tukey HSD *post-hoc* test. We compared the accuracies of the model training and model testing using a *t*-test for repeated measures. A confidence level of 5% was applied for the statistical comparisons.

## Results

### Inter-eye comparison of the retinal thickness for male and female subjects

To ensure that the selection of the eye did not introduce any bias, we conducted a comparison of the thickness of various retinal layers between the right and left eyes of participants of both sexes. Our analysis revealed that no significant differences were observed in any of the retinal layers between the eyes. Based on these findings, we opted to randomly select one eye from each participant for data extraction concerning retinal thickness. Table 1 displays the comparison of retinal thickness in the various datasets obtained from both eyes within the sample.

**TABLE 1:** Comparison of the retinal thickness obtained from both eyes of male and female groups.

Thickness (μm)	Male group		*p*-value	Female group		*p*-value
	Right eye	Left eye		Right eye	Left eye	
TR	323.6 ± 10.8	322.3 ± 10.8	0.65	311.1 ± 12.7	309.8 ± 13.4	0.72
RNFL	28 ± 2	27.3 ± 2.2	0.22	25.9 ± 2.3	25.3 ± 2	0.33
GCL	43.4 ± 2.4	43.3 ± 2.4	0.87	40.4 ± 3.6	40.3 ± 3.7	0.89
IPL	36.1 ± 1.6	36.1 ± 1.7	0.86	34 ± 2.7	34.2 ± 2.8	0.88
INL	35.6 ± 2.1	35.7 ± 2.1	0.91	33.5 ± 2.6	33.3 ± 2.3	0.79
OPL	28.4 ± 2.7	29.3 ± 2.8	0.23	28.8 ± 4.1	29.06 ± 4.1	0.81
ONL	69.2 ± 8.1	68.5 ± 8.3	0.76	66.6 ± 6.4	66.4 ± 7.1	0.90
RPE	15 ± 1.1	15 ± 1	0.93	14.9 ± 1.2	14.64 ± 1.4	0.45

TR, total retina; RNFL, retinal nerve fiber layer; GCL, ganglion cell layer; IPL, inner plexiform layer; INL, inner nuclear layer; OPL, outer plexiform layer; ONL, outer nuclear layer; RPE, retinal pigmented epithelium.

We randomly select one eye to extract retinal thickness and compared this feature between male and female groups, as depicted in Table 2. Our findings indicated significant differences in the total retina and layers comprising information from the inner retina (RNFL, GCL, IPL, INL), with the male group exhibiting greater thickness compared to the female group (*p* < 0.01). Conversely, no significant differences were discerned in the layers within the outer retina (OPL, ONL, RPE; *p* > 0.01).

**TABLE 2:** Comparison of retinal layer thickness between male and female groups.

	Retinal thickness (μm)		
Retinal layers	Male group	Female group	*p*-value
TR	322.9 ± 11.3	310.6 ± 12.9	0.0005
RNFL	27.8 ± 2.1	25.3 ± 1.8	<0.0001
GCL	43.2 ± 2.2	40.4 ± 3.5	0.0012
IPL	36 ± 1.6	34.1 ± 2.8	0.0039
INL	35.6 ± 2.2	33.4 ± 2.5	0.0016
OPL	29.1 ± 2.6	29.3 ± 4.2	0.78
ONL	68.7 ± 8.3	66.4 ± 6.8	0.28
RPE	15.1 ± 1	14.7 ± 1.2	0.22

TR, total retina; RNFL, retinal nerve fiber layer; GCL, ganglion cell layer; IPL, inner plexiform layer; INL, inner nuclear layer; OPL, outer plexiform layer; ONL, outer nuclear layer; RPE, retinal pigmented epithelium.

In the intergroup comparison, considering the thickness of different macular fields (Table 3), we observed that in datasets representing the total retina and data from the inner retina, the male group had thicker tissues across all fields than the female group (*p* < 0.01). However, in the datasets from the outer retina, we observed a predominance of non-significant differences.

**TABLE 3:** Comparison of retinal dataset thickness in the different macular fields from measurements obtained from both groups.

	Retinal thickness (μm)						
TR	Male	Female	*p*-value	RNFL	Male	Female	*p*-value
C	274.9 ± 15.9	258.6 ± 18.9	<0.01*	C	12 ± 1.5	10.3 ± 2.1	<0.01*
NI	353.1 ± 12.8	338.4 ± 16.9	<0.01*	NI	21.1 ± 2.1	18.8 ± 1.4	<0.01*
NO	328.8 ± 13.3	318 ± 14.0	0.04*	NO	48.6 ± 5.6	44.8 ± 4.5	<0.01*
TI	336.5 ± 10.3	320.6 ± 14.7	<0.01*	TI	16.9 ± 1.4	16 ± 0.9	<0.01*
TO	297.6 ± 13.9	283.3 ± 11.4	<0.01*	TO	18.8 ± 1.2	17.7 ± 1	<0.01*
SI	353 ± 12.8	340 ± 16.2	<0.01*	SI	24.5 ± 2.9	21.9 ± 2.4	<0.01*
SO	313.8 ± 13.3	305.7 ± 13.2	0.02*	SO	39.5 ± 5	36.6 ± 4.1	0.02*
II	351.1 ± 11.7	336.4 ± 16.8	<0.01*	II	26.1 ± 2.6	23.8 ± 2.2	<0.01*
IO	300.8 ± 10.8	291.9 ± 12.5	0.02*	IO	41.8 ± 4.8	39 ± 4.3	0.02*
**GCL**	**Male**	**Female**	***p*-value**	**IPL**	**Male**	**Female**	***p*-value**
C	15.4 ± 2.8	12.9 ± 4.1	<0.01*	C	21.2 ± 2.6	19.18 ± 3.1	<0.01*
NI	54.7 ± 3.9	50.7 ± 5.9	<0.01*	NI	45.2 ± 3	41.6 ± 4.7	<0.01*
NO	41.7 ± 3.5	40.7 ± 3.3	0.45	NO	31.9 ± 2.5	31.5 ± 3	0.51
TI	51.1 ± 3.4	46.3 ± 5.7	<0.01*	TI	43.7 ± 2.3	41.9 ± 4.3	0.02*
TO	41.2 ± 3.8	36.9 ± 3.5	<0.01*	TO	34.4 ± 2.6	32.3 ± 2.3	<0.01*
SI	55.4 ± 3.2	52.3 ± 5.7	0.01*	SI	44 ± 2.2	41.4 ± 3.4	<0.01*
SO	38.1 ± 2.8	37.3 ± 3.2	0.168	SO	31.1 ± 2.3	30.3 ± 2.5	0.15
II	55.5 ± 2.6	53.3 ± 5.3	0.02*	II	43.6 ± 1.9	41.2 ± 4.1	0.04*
IO	36.2 ± 3.2	35.3 ± 3.5	0.34	IO	29.5 ± 2	28.6 ± 2.5	0.21
**INL**	**Male**	**Female**	***p*-value**	**OPL**	**Male**	**Female**	***p*-value**
C	17.8 ± 4.1	13.9 ± 4.4	<0.01*	C	21.9 ± 4	22.1 ± 5.8	0.92
NI	41.59 ± 4	38.3 ± 4	<0.01*	NI	33 ± 6.5	32.1 ± 6.5	0.66
NO	37 ± 1.7	36.8 ± 2.8	0.4	NO	29.8 ± 3.1	29 ± 4.4	0.42
TI	37.7 ± 3.3	34.9 ± 3.4	0.01*	TI	29.3 ± 2.4	31.5 ± 7.1	0.20
TO	36.7 ± 2.2	34.8 ± 2.5	<0.01*	TO	27.3 ± 2	27.24 ± 3.8	0.95
SI	40.7 ± 3.2	39.3 ± 3.7	0.07	SI	32.4 ± 5.2	36.4 ± 9.5	0.11
SO	33.8 ± 2	32.9 ± 2.4	0.39	SO	27 ± 1.7	28.3 ± 4.8	0.23
II	42.2 ± 3.5	39.2 ± 4.3	<0.01*	II	32 ± 5.1	31.3 ± 5.4	0.46
IO	33.4 ± 2.3	31.8 ± 2.4	0.09	IO	26.8 ± 2.3	26.3 ± 2.8	0.23
**ONL**	**Male**	**Female**	***p*-value**	**RPE**	**Male**	**Female**	***p*-value**
C	93.6 ± 9	91 ± 11.63	0.43	C	18.3 ± 1.5	17.3 ± 1.7	0.21
NI	72.3 ± 13	71.1 ± 11.7	0.99	NI	16.2 ± 1.2	16 ± 1.6	0.92
NO	57.4 ± 8.8	58.7 ± 6.7	0.88	NO	14 ± 1.3	13.8 ± 1.4	0.77
TI	75.3 ± 7.8	71.1 ± 8	0.04*	TI	15.1 ± 1.3	14.4 ± 1.3	0.07
TO	60.8 ± 7.2	57.4 ± 6	0.09	TO	13.3 ± 1.2	13.2 ± 1.2	0.27
SI	71.3 ± 10.7	67.1 ± 11	0.07	SI	16 ± 1.5	15.7 ± 1.6	0.50
SO	64.1 ± 8.2	60.5 ± 6.8	0.15	SO	14.1 ± 1.2	13.6 ± 1.5	0.24
II	68.5 ± 10.3	68 ± 8.6	0.88	II	15.4 ± 1	15 ± 1.2	0.24
IO	54 ± 6.3	53 ± 5.5	0.68	IO	13.6 ± 1.2	13.1 ± 1.1	0.21

TR, total retina; RNFL, retinal nerve fiber layer; GCL, ganglion cell layer; IPL, inner plexiform layer; INL, inner nuclear layer; OPL, outer plexiform layer; ONL, outer nuclear layer; RPE, retinal pigmented epithelium. C, central retina; NI, nasal inner; NO, nasal outer; TI, temporal inner; TO, temporal outer; SI, superior inner; SO, superior outer; II, inferior inner; IO, inferior outer.

*p < 0.05.

### Machine learning accuracies during model training

Table 4 presents the mean accuracies (± standard deviation) derived from model training across various classifiers and retinal datasets. The results of a two-way ANOVA revealed significant effects attributed to both the algorithm factor, the retinal dataset factor, and the interaction between these two factors, as summarized in Table 5. Notably, *post hoc* multiple comparisons demonstrated that the accuracies achieved by all algorithms were markedly superior when utilizing the total retina dataset and datasets originating from the inner retina (RNFL, GCL, IPL, INL), as compared to datasets from the outer retina (OPL, ONL, and RPE).

**TABLE 4:** Comparison of mean accuracies (± standard deviation) obtained from the machine learning algorithms to classify the sex-related differences in the retinal layers (and total retina) for model training.

	Machine learning algorithm accuracies (%)						
Layers	SVC	GNB	RF	KNN	LR	LD	DT
RPE	72.5 ± 9.6	71.8 ± 5.1	83 ± 4.5	64.3 ± 5.5	68.5 ± 6.2	69.3 ± 7.6	71.3 ± 9.9
ONL	69.3 ± 6.5	67.8 ± 7.1	90.5 ± 5.5	68.5 ± 9.4	67.5 ± 8.7	65.3 ± 6.3	82.8 ± 5.2
OPL	79.8 ± 7.5	70.5 ± 4.1	89.5 ± 5.8	71.8 ± 5	65.5 ± 7.5	66.8 ± 7.8	79.8 ± 6.8
INL	84.5 ± 4.5	89 ± 3.4	92.3 ± 3.8	81.5 ± 6.5	85 ± 6.2	85.8 ± 6.7	81.5 ± 6
IPL	85.8 ± 3.3	88 ± 3.3	92 ± 5	83.8 ± 5.4	84.5 ± 6.1	82.3 ± 5.3	82.3 ± 7.2
GCL	89.5 ± 3.7	90 ± 4.3	94.5 ± 2	89.8 ± 4.3	85.5 ± 4.7	87.5 ± 4.3	88 ± 3.9
RNFL	92.3 ± 2.8	91.5 ± 3.8	94.5 ± 2.8	91 ± 4.6	90.5 ± 3.9	91 ± 3.2	90.8 ± 4.7
TR	92.5 ± 2.3	96 ± 2.4	96.5 ± 3.9	91 ± 4.6	93.5 ± 5.3	93.3 ± 3.9	89 ± 4.3

TR, total retina; RNFL, retinal nerve fiber layer; GCL, ganglion cell layer; IPL, inner plexiform layer; INL, inner nuclear layer; OPL, outer plexiform layer; ONL, outer nuclear layer; RPE, retinal pigmented epithelium; SVC, support vector classification; GNB, Gaussian Naïve Bayes; RF, random forest; kNN, k-nearest neighbors; LR, logistic regression; LD, linear discriminant; DT, decision tree.

**TABLE 5:** Two-way ANOVA results for model training.

	SS	DF	MS	F	*p*-value
Algorithms	7,937	6	1,322.9	42.87	< 0.001
Retinal datasets	37,140	7	5,305.7	171.93	< 0.001
Algorithms*retinal datasets	6,703	42	159.6	5.17	< 0.001
Residuals	15,554	504	30.9		

SS, sum of squares; DF, degrees of freedom; MS, mean squares.

In evaluating the accuracies of different algorithms across the diverse retinal datasets, multiple comparisons indicated a notable absence of significant differences in algorithm performance within the total retina dataset and the inner retina datasets (*p* > 0.05). Conversely, in the OPL dataset, it was evident that random forest (RF), support vector classifier (SVC), and decision tree (DT) exhibited significantly higher accuracies when contrasted with other algorithms. Similarly, in the ONL dataset, random forest and decision tree outperformed their counterparts. Notably, in the RPE dataset, random forest demonstrated the highest accuracy among all algorithms.

### Machine learning accuracies during model testing

Table 6 displays the mean accuracies (± standard deviation) derived from model testing across various classifiers and retinal datasets. Once again, the results of a two-way ANOVA revealed significant effects associated with the algorithm factor, the retinal dataset factor, and their interaction (as summarized in Table 7). *Post hoc* multiple comparisons further substantiated that, much like the training model, all algorithms achieved significantly higher accuracy levels when employing the total retina dataset and datasets from the inner retina, in comparison to the datasets from the outer retina. Consistent with the training model, the results of multiple comparisons within the total retina dataset and datasets from the inner retina indicated an absence of significant differences in algorithm accuracies (*p* > 0.05). In contrast, concerning the outer retina, random forest (RF) exhibited notably higher accuracy compared to other algorithms (*p* < 0.05).

**TABLE 6:** Comparison of mean accuracies (± standard deviation) obtained from the machine learning algorithms to classify the sex-related differences in the retinal layers (and total retina) for model testing.

	Machine learning algorithms (%)						
Layers	SVC	GNB	RF	KNN	LR	LD	DT
RPE	68.8 ± 9.5	70.8 ± 5.8	76.8 ± 3.7	59.8 ± 5.2	66 ± 5.6	66.5 ± 7.3	69.5 ± 6
ONL	70.5 ± 8.8	63.5 ± 6.7	89.3 ± 5.4	68 ± 6.5	64.3 ± 5.9	66.8 ± 10	75.5 ± 6
OPL	76.3 ± 4.3	74.5 ± 7.9	88.5 ± 3.8	72 ± 5.2	64.5 ± 9	62.8 ± 7.2	83 ± 6.2
INL	82.8 ± 6.3	85.8 ± 5.5	88.8 ± 4	80.5 ± 6.3	77.5 ± 6.6	80.8 ± 5.3	85.3 ± 7
IPL	85.3 ± 6.6	83.3 ± 5.5	93.8 ± 2.4	80.5 ± 5.1	82.3 ± 5.8	84.3 ± 4.3	85.8 ± 7.1
GCL	89.3 ± 4.4	82 ± 4.1	92.3 ± 4.2	84 ± 7.4	86.3 ± 5.3	88.8 ± 5	89.8 ± 4.4
RNFL	91.5 ± 3.8	91 ± 3.9	94.5 ± 4.7	92.3 ± 4.6	90.3 ± 5.1	91.3 ± 4.6	90 ± 5.3
TR	91.8 ± 2.7	93.3 ± 3.7	94 ± 4.1	89.5 ± 5.1	91.3 ± 3.6	91.5 ± 5.7	91.3 ± 4.3

TR, total retina; RNFL, retinal nerve fiber layer; GCL, ganglion cell layer; IPL, inner plexiform layer; INL, inner nuclear layer; OPL, outer plexiform layer; ONL, outer nuclear layer; RPE, retinal pigmented epithelium; SVC, support vector classification; GNB, Gaussian Naïve Bayes; RF, random forest; kNN, k-nearest neighbors; LR, logistic regression; LD, linear discriminant; DT, decision tree.

**TABLE 7:** Two-way ANOVA results for model testing.

	SS	DF	MS	F	*p*-value
Algorithms	8,299	6	1,383.1	41.91	<0.001
Retinal datasets	41,178	7	5,882.6	178.25	<0.001
Algorithms*retinal datasets	6,276	42	149.4	4.53	<0.001
Residuals	16,633	504	33		

SS, sum of squares; DF, degrees of freedom; MS, mean squares.

### Comparison of the accuracies estimated for the models in the training and testing stages

**FIGURE 1:**
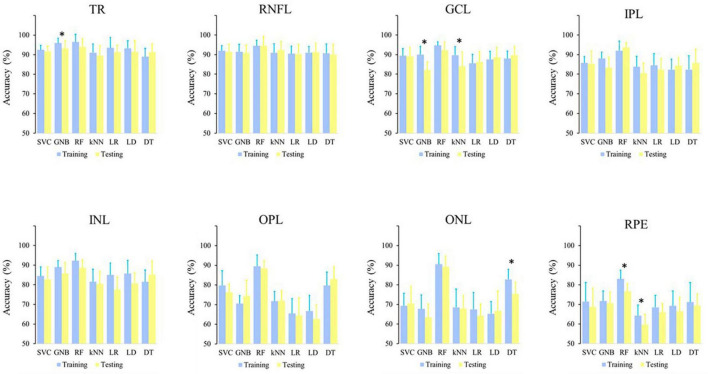
Comparison of the algorithm accuracies calculated in the model training and model testing in the different retinal datasets. *p < 0.05.

The comparison of the accuracies calculated for the models in the training and testing showed that 10.8% of the comparisons had significant differences, and all of them showed higher accuracy of the model in the training (Figure 1).

**FIGURE 2:**
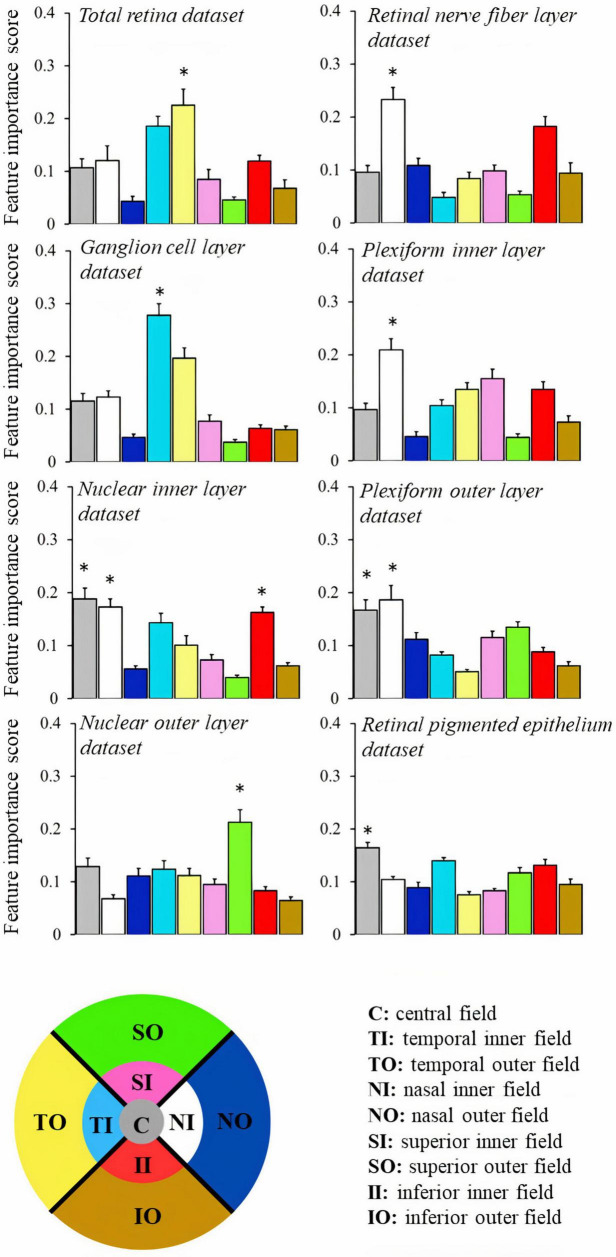
Comparison of the feature importance score obtained from random forest algorithm to classify the sex of the participant based on retinal thickness from different datasets. The color code is indicated at the bottom of the figure. *p < 0.05.

After finding that the random forest classifier outperformed other methods in classifying the datasets, we examined feature importance scores, which indicate the extent to which each feature influences the model’s predictions. Random forest employs the Gini impurity, which reveals how frequently a feature is used to split the data in its decision trees. Figure 2 displays the feature importance scores for macular thickness in different fields. We conducted one-way ANOVA to assess the impact of the macular field on the feature importance score for each dataset. We found that in all datasets, there were significant differences (*p* < 0.01), with one or more fields having a greater importance than others in the classification decision.

## Discussion

This study’s findings reveal significant patterns in the classification accuracy of sex-specific data, utilizing various retinal layers and ML algorithms. The most reliable accuracies for accurately distinguishing between male and female participants were observed when analyzing data from the total retinal structure and the retinal nerve fiber layer. These results suggest that these retinal layers possess unique sex-related characteristics that were effectively identified by the employed ML techniques. Interestingly, the highest classification accuracies were consistently achieved using these retinal layers, yet no statistically significant differences were detected among the accuracies derived from the various ML algorithms used in this study. This suggests that the algorithms consistently performed when tasked with sex classification based on retinal data, regardless of their inherent methodologies. Moreover, a fascinating trend was observed where classification accuracies showed a decreasing trend as the analysis moved toward the outer retinal layers. Additionally, some algorithms demonstrated statistically significant deviations from others in terms of classification accuracy. Notably, the RF algorithms displayed higher accuracies compared to the others in this context.

While the sex of a patient is typically known during a consultation, it is not always evident whether the retinal thickness of that patient aligns with the sex-based patterns expected. Comparing a patient’s retinal thickness to sex-based populational norms can be a valuable tool in evaluating the patient. However, alternative approaches, such as machine learning, can complement conventional statistical methods. For instance, our study revealed that, even in retinal layers where there were no significant differences in thickness between the male and female groups, such as the datasets from the outer retina, we achieved a sex classification accuracy exceeding 75%. What would it signify if a male patient were classified as female based on retinal thickness patterns, or vice versa? It is crucial to emphasize that this classification does not pertain to the patient’s actual sex but rather reflects the retinal thickness patterns expected for each sex. The clinical implications of a disparity between a patient’s actual sex and a different sex classification based on retinal structure remain unclear, but further investigations may shed light on this question.

An investigation has previously been conducted using a deep learning method to predict sex through macular OCT images (24). It showed that the differences between male and female subjects might not be uniform throughout the macula. The best accuracy in separating data from male and female subjects occurred in the central fovea (around 75%) and lower accuracy was found in the external limit of the fovea (around 70%). They also fed models considering different macular sectors and found non-uniformity in the accuracies (ranging between 52 and 62%). The data they used are comparable to the total retina dataset of the present study. We interpreted that our accuracies were higher because we had fed our models with thickness information of all the macular sectors, and they used information from each sector for their classification. Taking into account the significance of macular field thickness, our results align with the findings achieved using deep learning approaches for the total retinal dataset, wherein the temporal fields were identified as the most crucial for classifying sex. The current study also revealed that in other retinal layers, the field of greatest importance varied.

The difference between the accuracies of the training and testing models is a crucial aspect in the evaluation of machine learning models. This difference can provide insights into how well the model is generalizing to unseen data, which is essential for determining the model’s robustness. In the current study, the vast majority of comparisons showed no significant discrepancy between the training and testing accuracies, which is a positive indication. It suggests that the model, which fits the training data well, also exhibits good generalization to new data. This alignment between training and testing accuracies indicates that the model is not overfitting the training data and has the potential for reliable performance on new, unseen data.

The superior performance of random forest in achieving higher accuracies compared to alternative machine learning algorithms in our study can be attributed to several key advantages of this ensemble learning technique. random forest harnesses the power of multiple decision trees, where each tree is trained on a different subset of the data and with feature randomness (25). This inherent diversity and randomness help mitigate overfitting, a common challenge in machine learning, by reducing the model’s sensitivity to noise and outliers (26). Moreover, random forest’s ability to handle both classification and regression tasks, its capacity to capture complex non-linear relationships in the data, and its robustness to multicollinearity make it particularly well-suited for a wide range of datasets (27). Additionally, the ensemble nature of random forest allows it to aggregate the predictions from multiple trees, reducing the risk of bias that can be associated with individual models. Consequently, the comprehensive nature of random forest, combining predictive power and robustness, positions it as an attractive choice for achieving high accuracy in diverse machine learning tasks.

Prior research has suggested that male participants typically display a greater retinal thickness compared to female participants (7–12). The impact of sex on retinal layers is still a topic of ongoing debate. Some studies (13–17) have reported thicker retinal layers in male subjects (GCL, IPL, INL, OPL, and ONL), while others have observed minimal or no sex-related differences (18, 19). Some studies have shown that female subjects had a thicker peripapillary RNFL than male subjects (28, 29). The present study uncovers a greater thickness in the inner retinal layers of male subjects compared to female subjects. Sexual hormones interacting with receptors such as estrogen and androgen receptors can affect ocular tissue. However, despite their influence on various ocular structures, the effect of these hormones on retinal layer thickness remains largely uninvestigated (30–35).

Neglecting to account for sex differences in comparisons of retinal thickness between healthy individuals and patients could result in erroneous diagnoses, particularly for inner retinal diseases that display substantial sex-related disparities. Conditions like glaucoma, macular holes, diabetic retinopathy, and age-related macular degeneration demonstrate varying prevalence rates between male and female subjects. This is likely attributable to changes in sex hormone concentrations after the age of 50 (36, 37).

The current investigation focuses on recruiting predominantly young adult participants, and as a result, the applicability of our findings may be limited to this specific age group. This demographic constraint represents a notable limitation of our study. To enhance the generalizability and robustness of our conclusions, it is imperative for future research endeavors to encompass a broader spectrum of cases, incorporating individuals from various age ranges. In the present study, our primary aim was to demonstrate that various models can learn pertinent sex-related patterns within diverse retinal datasets. While the current sample size has proven adequate for this initial validation, it remains a limitation of the study and should be expanded in future research endeavors.

In conclusion, this research highlights the discriminative capacity of different retinal layers in sex classification, achieving varying levels of accuracy across distinct layers and ML algorithms. The consistently superior performance of the RF algorithm indicates its effectiveness in identifying sex-related characteristics in various retinal layers. Furthermore, the identified patterns of accuracy fluctuations across retinal layers offer invaluable insights for subsequent research and algorithmic advancement in the field of retinal data analysis.

## Data availability statement

The original contributions presented in this study are available after inquiries directed to the corresponding author.

## Ethics statement

The studies involving humans were approved by the Ethical Committee for Research in Humans of the Universidade Federal do Pará (report number 3.285.557). The studies were conducted in accordance with the local legislation and institutional requirements. The participants provided their written informed consent to participate in this study.

## Author contributions

FF: Conceptualization, Formal analysis, Investigation, Methodology, Writing – original draft, Writing – review & editing. RS: Conceptualization, Supervision, Writing – review & editing. ER: Formal analysis, Software, Writing – review & editing. AS: Investigation, Writing – review & editing. GSAS: Investigation, Writing – review & editing. AR: Investigation, Writing – review & editing. MC: Formal analysis, Funding acquisition, Writing – review & editing. GSS: Conceptualization, Data curation, Funding acquisition, Methodology, Project administration, Software, Supervision, Writing – original draft, Writing – review & editing.
